# Age-related clinical characteristics of children and adolescents with ADHD

**DOI:** 10.3389/fpsyt.2023.1069934

**Published:** 2023-01-27

**Authors:** Pietro De Rossi, Barbara D’Aiello, Italo Pretelli, Deny Menghini, Silvia Di Vara, Stefano Vicari

**Affiliations:** ^1^Child and Adolescent Neuropsychiatry Unit, Department of Neuroscience, Bambino Gesù Children’s Hospital, Istituto di Ricovero e Cura a Carattere Scientifico, Rome, Italy; ^2^Department of Human Sciences, Libera Università Maria Santissima Assunta University, Rome, Italy; ^3^Department of Life Sciences and Public Health, Università Cattolica del Sacro Cuore, Rome, Italy

**Keywords:** hyperactivity, children, adolescents, emotional dysregulation, functional impairment, anxiety, depression, ADHD symptoms

## Abstract

**Introduction:**

Attention deficit hyperactivity disorder (ADHD) has been associated with difficulties in regulating aversion states, high functional impairment, and a high risk of psychopathology across the lifespan. ADHD is clinically heterogeneous, with a wide spectrum of severity and associated symptoms. Clinical characteristics need to be carefully defined in different periods of life as ADHD course, symptoms, and comorbidities may fluctuate and change over time. Adolescence usually represents the transition from primary to secondary education, with a qualitative and quantitative change in environmental and functional demands, thus driving symptoms’ change.

**Methods:**

In order to characterize age-related clinical features of children (<11 years) and adolescents (≥11 years) with ADHD, we conducted a naturalistic study on 750 children and adolescents assessed for ADHD at our Neuropsychiatry Unit over the course of 3 years (2018–2020).

**Results:**

We found that ADHD symptoms were significantly higher in children than adolescents. More importantly, we found worse global functioning, lower adaptive skills, higher levels of anxiety and depressive symptoms, somatic complaints, emotional dysregulation, social problems, and aggression in adolescents, despite a lower severity of ADHD-specific symptoms.

**Conclusion:**

These results should be confirmed in longitudinal observational studies of adequate sample size in order to reliably describe a potential course characterized by worsening of functioning, reduction in ADHD-specific symptoms and increase in general psychopathology during the transition from childhood to adolescence.

## 1. Introduction

Attention deficit and hyperactivity disorder (ADHD) is one of the most common neurodevelopmental disorders diagnosed in early childhood and adolescence. According to DSM-5, it is characterized by altered and unusual levels of inattention and hyperactivity compared to those observed in typical child development (1). ADHD symptoms frequently determine significant functional impairment in familiar, academic, and social context. Moreover, it is usually diagnosed in scholar age, with a subsequent course that is often characterized by progressively overlapping comorbid disorders (2). Finally, it is well-established that methylphenidate (MPH) is the first line pharmacological treatment for this disorder, with significant positive effects on the improvement of attention, organization, hyperactivity, and impulse control (3). This is worth mentioning as it has been showed that a timely and proper multidimensional treatment of ADHD can result in the prevention of unfavorable developmental trajectories including deviant behaviors and substance abuse (4).

Attention deficit hyperactivity disorder worldwide prevalence in school-age children is 5.3% (5, 6), with a male-to-female ratio of 3:1 in population-based studies and between 5:1 and 9:1 in clinical samples (7). It has been confirmed that 66.3% of the diagnosed children and adolescents assume medication for the disorder, representing 4.8% of all children aged 4–17 years. In Italy, a prevalence range between 1.1 and 3.1% is estimated among children and adolescents aged 5 and 17 years, with boys displaying a prevalence rate 1.2–7.6 higher than girls (8). It may be noticed that Italian prevalence is lower than the estimated worldwide prevalence, and this is probably due to methodological and cultural factors that are ad-dressed within the Italian prevalence study (8).

In two recent papers we have described clinical characteristics of children and adolescents with ADHD on the base of their selection for first MPH prescription and gender (9, 10). The rationale of these descriptive studies was to provide clinicians with information potentially helpful in treatment personalization after diagnostic assessment. There is substantial evidence that children with a diagnosis of ADHD show some symptom improvement during adolescence, particularly as regards hyperactivity/impulsivity, but also retain ADHD symptoms associated with functional impairment and show a higher risk of substance use disorders and mood disorders (2, 11, 12). This is also consistently observed in daily clinical practice. Furthermore, it is not unusual that patients, especially if ADHD is not severe, are referred for diagnostic assessment or treatment around or after 11 years of age (13). This age cut-off usually represents the transition from primary education to secondary education, with qualitative change and quantitative increase in environmental and functional demands in both academic and extra-academic context.

Here we propose a cross-sectional study defining clinical characteristics of children and adolescents with ADHD according to their age. Specifically, we carried out a comparison of psychopathological variables, clinical severity, and functioning measures on a large sample of children and adolescents referred for assessment at our child and adolescent Neuropsychiatry Unit, after a dichotomization into two age groups: patients aged < 11 (ADHD/primary school) and patients with ADHD aged ≥ 11 (ADHD/secondary school).

Our main hypothesis was that older patients would display a higher severity of mood-related symptoms and other non-ADHD-specific symptoms, along with higher levels of global functional impairment. Such descriptive characterization could significantly help clinicians personalize treatment strategies after diagnostic assessment.

## 2. Materials and methods

### 2.1. Participants

In this observational cross-sectional study, 750 children and adolescents with ADHD who attended the Child and Adolescents Neuropsychiatry Unit of the Bambino Gesù Children’s Hospital (Rome, Italy) were recruited over the course of 3 years, from 2018 to 2020. We included only children and adolescents with 1) an ADHD diagnosis accordingly to the Diagnostic and Statistical Manual of Mental Disorders, Fifth Edition—DSM-5 (1); (2) an intelligence quotient (IQ) of 85 or higher. We excluded children and adolescents with (1) the presence of Autism Spectrum Disorders or psychiatric disorders (i.e., Schizophrenia Spectrum Disorders, or Post Traumatic Stress Disorder) as comorbid conditions; (3) a history of neurological or medical or genetic conditions. Patients of all ages were referred on the base of a suspected ADHD diagnosis due to functioning problems associated to inattentive and hyperactive symptoms.

Children and adolescents (Mean age = 9.68, SD = 2.98; 99 females/651 males) received their diagnosis of ADHD from experienced developmental psychiatrists and neuropsychologists according to the Diagnostic and Statistical Manual of Mental Disorders (DSM-5) criteria (1).

In line with the aim of the current study, the sample was divided into two groups: 513 children with ADHD aged < 11 (ADHD/primary school) and 237 adolescents with ADHD aged ≥ 11 (ADHD/secondary school). The division of its groups at age 11 was established considering that in Italy the elementary school cycle ends at about age 10 and the first cycle of secondary school is started at age 11.

Considering our sample, 7.87% were Inattentive, 0.93% Hyperactive/Impulsive, 87.33% Combined, and 3.87% ADHD-Not Otherwise Specified (NOS).

Psychiatrists and neuropsychologists collaborated in the diagnosis of each case. Psychiatric diagnoses were based on developmental history, extensive clinical examination, and The Kiddie Schedule for Affective Disorders and Schizophrenia for School Aged Children Present and Lifetime Version DSM-5 (14), a semi-structured interview that assesses the presence of psychopathological disorders according to DSM-5 classification. The majority of items are scored using a 0–3 point rating scale. Scores of 0 indicate no information is available; scores of 1 suggest the symptom is not present; scores of 2 indicate sub-threshold presentation, and scores of 3 indicated threshold presentation of symptoms. Five groups of psychiatric comorbidities were identified as follows: Mood Disorders (0.5%, including Depressive Disorders and Bipolar Disorders), Behavioral Disorders (17.07%, including Conduct Disorder, and Oppositional Defiant Disorder), Learning Disorders (16%), Anxiety Disorders (3.8% including Separation Anxiety, Generalized Anxiety Disorder and Obsessive-Compulsive Disorder), and Generalized Developmental Delay (15.5%, including communication atypia, Speech Disorders, Developmental Coordination Disorder). Moreover, the number of patients who received a comorbid psychiatric diagnosis was considered.

All participants and parents were informed about assessment instruments and treatment options. The study conformed to the Declaration of Helsinki.

### 2.2. Instruments

The data used for the purpose of analysis were obtained retrospectively from the patients’ medical records. The diagnostic evaluation performed at our facility follows a standard procedure: children/adolescents are evaluated on three consecutive days. On the first day, the medical history is collected, and the children/adolescents are tested for cognitive functioning to rule out comorbidity with intellectual disability. Parents fill out several questionnaires to investigate adaptive functioning and emotional and behavioral symptoms. On the second day, a semi-structured interview is administered to children and their parents in order to assess psychopathological aspects and, the clinician completes the impairment scale of functioning. On the third day, children/adolescents undergo a learning/language assessment to rule out other comorbid disorders (data from the third-day evaluation were not included in this study).

To evaluate the global functioning the Children’s Global Assessment Scale (C-GAS) (15) was administered. The C-GAS considers a range of overall disorder severity from 0 to 100. Scores below 70 indicate impaired global functioning.

To evaluate IQ, the Perceptual Reasoning Index of the Wechsler Intelligence Scale for Children-IV (16) or Colored Progressive Matrices or Standard Progressive Matrices (17) were considered. The global IQ was considered in the analysis (*M* = 100, SD = 15).

To assess adaptive skills the questionnaire was administered to parents, namely the Adaptive Behavior Assessment System (ABAS-II) (18). The standard General Adaptive Composite (GAC) score (M = 100, SD = 15) was considered in the analysis.

For the purpose of investigating ADHD symptoms, parents completed the questionnaire Conners’ Parent Rating Scales Long Version Revised–CPRS-R:L (19). The CPRS-R:L is a questionnaire that is completed by parents to obtain a measure of ADHD hyperactivity and inattention symptoms across 14 subscales. It generates a T-score for each subscale. We analyzed the following 2 subscales: DSM-IV Inattentive and DSM IV Hyperactive/Impulsive. T-scores > 70 were classified as clinically relevant, T-scores between 60 and 69 were classified as borderline, and T-scores < 60 indicated non-clinical symptoms. T-scores were used for statistical analysis.

To assess behavioral and emotional symptoms the Achenbach System of Empirically Based Assessment (ASEBA) questionnaire was filled out by parents. The CBCL parent questionnaire (20) is a well-known tool to detecting psychopathological symptoms in children and adolescents. The hierarchical structure of the CBCL includes 113 items and several scales. It generates a T score for each subscale. We analyzed the following syndromic scales (Anxiety/Depression, Somatic Disorders, Social Problems, Attention Problems, and Aggressive Behavior) as these scales do not contain overlapping items. According to the cut-off thresholds of Achenbach and Rescorla (15), T-scores > 70 were classified as clinically relevant, T-scores between 60 and 69 were classified as borderline, and T-scores < 60 indicated non-clinical symptoms. T-scores were used for statistical analysis.

The dysregulation profile (DP) of CBCL, characterized by simultaneous high values (greater than two standard deviations) in three syndromic scales (anxiety/depression, attention problems, and aggressive behavior), was calculated using the sum of the T-scores of the three syndromic scales. Scores ≥ 210 are considered clinically significant, those between 180 and 209 are in the borderline range, and those ≤ 179 are non-clinical scores.

### 2.3. Statistical analyses

The Shapiro–Wilk test was used to test the normality of the data and Levene’s test for the homogeneity of variances. Non-parametric tests were computed when data were not normally distributed, and the assumption of homogeneity was violated (see Section “3. Results”). Specifically, for continuous variables Mann-Whitney *U* Test was used to compare the two groups on age, IQ, ABAS, C-GAS, and DP because the assumption of normality was not fulfilled. Kruskal–Wallis ANOVA was conducted to compare the two groups on CPRS-R:L Scales and CBCL Scales. Bonferroni’s correction for multiple comparisons was applied. For categorical variables, non-parametric analyses were conducted. Specifically, Chi-square analyses were run to test differences between the two groups on gender, ADHD presentation, and drug use. *Post-hoc* comparison procedures for interpreting Chi-square contingency-table test results were conducted in line with Beasley and Schumacker (21).

## 3. Results

### 3.1. Results on demographic variables

As expected, the two groups differed for age (Z = −22.03, *p* < 0.0001, Cohen’s *d* = 2.70; respectively, *M* = 7.99, SD = 1.57; *M* = 13.35, SD = 1.27) but did not differ for gender [χ^2^_(1)_ = 1.50, *p* = 0.22] and IQ [Z = −0.58, *p* = 0.55; respectively, ADHD/primary school: M = 104.27, SD = 17.64; ADHD/secondary school: M = 104.24, SD = 19.96]. The two groups differed for ADHD presentation [χ^2^_(3)_ = 20.33, *p* < 0.0001]. *Post-hoc* comparison documented that ADHD/primary school group showed a higher number of children with Hyperactive/Impulsive (*p* < 0.0001) and NOS (*p* < 0.0001) than ADHD/secondary school group. Moreover, the two groups did not differ for psychiatric comorbidities [χ^2^_(5)_ = 3.23, *p* = 0.66]. Out of 750 patients, 2% (15 patients) were already in treatment with MPH while 40.7% (305 patients) had a first prescription of MPH after diagnostic assessment. ADHD/primary school and ADHD/secondary school differed for ongoing MPH [χ^2^_(1)_ = 8.76, *p* = 0.03] and for first MPH prescription [χ^2^_(1)_ = 4.74, *p* = 0.02] (see Table 1).

**TABLE 1:** Demographic information of ADHD/primary school and ADHD/secondary school groups.

	ADHD/primary school N (%)	ADHD/secondary school N (%)
**Gender**		
Male	440 (85.77)	211 (89.03)
Female	73 (14.23)	26 (10.97)
**ADHD presentation**		
Inattentive	27 (5.2)	32 (13)
Hyperactive/Impulsive	7 (1.4)**	0 (0)
Combined	455 (88.7)	200 (84.4)
NOS	24 (4.7)**	5 (2.6)
**Comorbid psychiatric diagnosis**		
Mood disorders	3 (0.58)	1 (0.42)
Behavioral disorders	96 (18.71)	40 (16.88)
Learning disorders	81 (15.79)	42 (17.72)
Anxiety disorders	19 (3.7)	10 (4.22)
Generalized developmental delay	89 (17.35)	30 (12.26)
No comorbidity	225 (43.87)	114 (48.5)
**MPH treatment**		
Ongoing	5 (0.97)*	10 (4.22)
First prescription	195 (38.02)*	110 (46.41)

*p < 0.05; **p < 0.001; Significant difference compared to ADHD/secondary school group.

### 3.2. Results on global functioning

**FIGURE 1:**
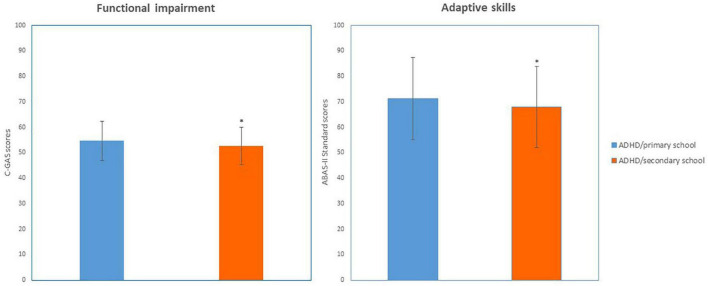
Functioning (C-GAS and ABAS-II scores) in ADHD/primary school and ADHD/secondary school groups. *p < 0.05; Significant difference compared to ADHD/primary school group.

The two groups differed for functional impairment derived from C-GAS scores (*Z* = 3.02, *p* = 0.002, Cohen’s *d* = 0.26) and for adaptive skills derived from ABAS-II scores (*Z* = 2.47, *p* = 0.013, *p* = 0.01, Cohen’s *d* = 2.71) (see Figure 1 and Table 2).

**TABLE 2:** Results of comparison between the two groups on global functioning.

	ADHD/primary school	ADHD/secondary school
	Mean (SD)	Mean (SD)
ABAS	71.28 (16.08)	68.01 (15.87)*
C-GAS	54.67 (7.72)	52.66 (7.31)*

*p < 0.05; Significant difference compared to ADHD/primary school group.

### 3.3. Results on psychopathological symptoms

**FIGURE 2:**
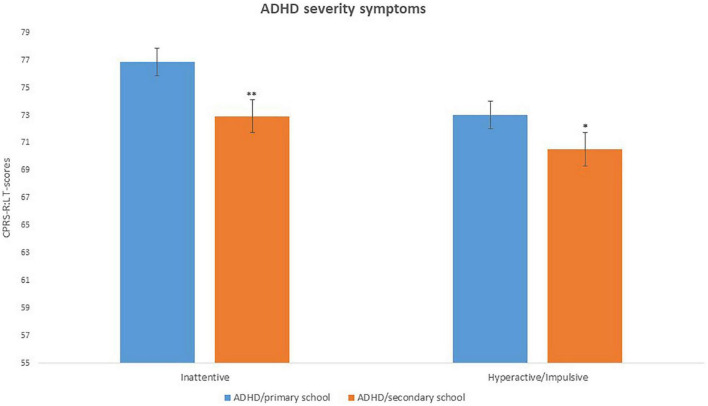
ADHD severity of symptoms (CPRS-R:L scores) in ADHD/primary school and ADHD/secondary school groups. *p < 0.05; **p < 0.001; Significant difference compared to ADHD/primary school group.

Concerning severity of ADHD symptoms, Kruskal–Wallis ANOVA on ADHD symptoms was conducted with 2 CPRS-R:L subscales (DSM-IV Inattentive and DSM IV Hyperactive/Impulsive). Results on the DSM-IV Inattentive subscale revealed a significant difference [*H*_(718)_ = 17.16, *p* < 0.0001, η^2^ = 0.004], with mean scores of ADHD/primary school group higher (*M* = 76.85, SD = 13.47) than those of ADHD/secondary school group (*M* = 72.94, SD = 12.31). Results on the DSM-IV Hyperactive/Impulsive subscale revealed a significant difference [*H*_(718)_ = 4.34; *p* = 0.03, η^2^ = 0.15] with mean scores of ADHD/primary school group higher (*M* = 73.03, SD = 13.36) than those of ADHD/secondary school group (*M* = 70.52, SD = 14.59) (see Figure 2 and Table 3).

**TABLE 3:** Results of comparison between the two groups on psychopathological symptoms.

	ADHD/primary school	ADHD/secondary school
	Mean (SD)	Mean (SD)
CPRS-R:L Inattentive	76.85 (13.47)	72.94 (12.31)**
CPRS-R:L Hyperactive/Impulsive	73.03 (13.36)	70.52 (14.59)*
CBCL Anxious/Depressed	62.77 (9.07)	65.94 (9.42)**
CBCL Somatic complaints	58.33 (7.75)	61.24 (8.38)**
CBCL Social problems	64.10 (8.53)	66.68 (9.05)**
CBCL Attention problems	69.64 (9.32)	69.31 (8.81)
CBCL Aggressive behavior	66.00 (10.58)	68.39 (10.17)*
CBCL DP	198.16 (23.38)	203.65 (23.11)*

*p < 0.05; **p < 0.001; Significant difference compared to ADHD/primary school group.

**FIGURE 3:**
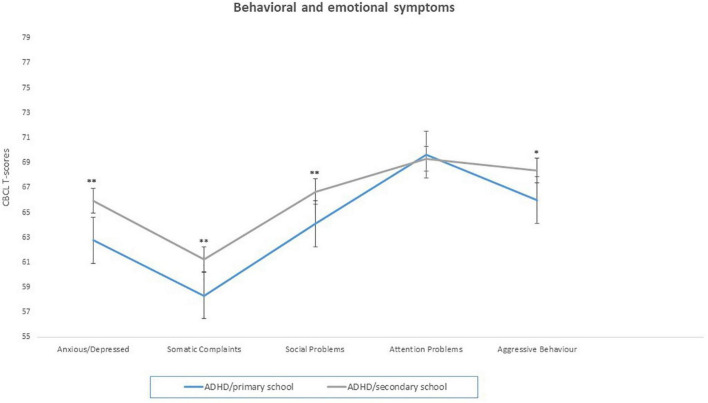
Behavioral and emotional symptoms (CBCL scores) in ADHD/primary school and ADHD/secondary school groups. *p < 0.05; **p < 0.001; Significant difference compared to ADHD/primary school group.

Differences between ADHD/primary school group and with ADHD/secondary school group in behavioral and emotional symptoms, as measured by the CBCL questionnaire, were investigated. Kruskal–Wallis ANOVA, was conducted on 5 CBCL subscales (Anxious/Depressed, Somatic Complaints, Social Problems, Attention Problems, and Aggressive Behavior) as within factor and Group (ADHD/primary school vs. ADHD/secondary school) as between factors. Results documented significant differences between the two group in several CBCL subscales, as Anxious/Depressed [*H*_(715)_ = 16.55, *p* < 0.0001, η^2^ = 0.02], Somatic Complaints [*H*_(715)_ = 23.09, *p* < 0.0001, η^2^ = 0.03], Social Problems [*H*_(715)_ = 11.64, *p* = 0.0006, η^2^ = 0.01], Aggressive Behavior [*H*_(714)_ = 8.68, *p* = 0.003, η^2^ = 0.01], but not Attention Problems [*H*_(714)_ = 0.51, *p* = 0.47]. DP scores also differed between groups [*Z* = −2.72, *p* = 0.006, Cohen’s *d* = 0.23] (see Figure 3 and Table 3).

## 4. Discussion

This observational cross-sectional study investigated age-related clinical characteristics on a group of 750 children and adolescents at their first diagnostic assessment for ADHD at our center.

On the epidemiological level, our results were in line with international literature (22), with a male to female ratio of approximately 6:1. Coherently, boys are more likely to be referred, diagnosed and treated for ADHD symptoms than girls (9).

The two groups did not differ for gender and IQ but differed for ADHD presentation as the ADHD/primary school group showed a higher number of children with Hyperactive/Impulsive presentation and ADHD NOS than the ADHD/secondary school group. This is consistent with the observation that younger patients at their first assessment may present more frequently with less specific clinical pictures. Furthermore, at younger ages, functional and academic demands may be less challenging thus keeping some compensated symptoms below the diagnostic threshold. Finally, it is rather typical that hyperactive/impulsive symptoms are significantly more pronounced before pre-pubertal age, with a progressive decline from adolescence to adulthood (23).

Our first main finding is that the two groups differ for functional impairment, and adaptive skills. This is in line with previous evidence that, despite the presence of comorbidity, early ADHD symptoms are among the most important risk factors for impaired daily functioning in adolescence (24). In particular, we observed poorer functioning and lower adaptive skills in older patients despite significantly less severe ADHD-specific symptoms and no significant differences in IQ. This finding could be interpreted at least in part as a consequence of pre-existing, more severe symptoms that prevented an adequate acquisition of basic skills required to develop further functional autonomy from childhood to adulthood (11). An alternative explanatory hypothesis for functional worsening could be that increased functional demands in academic or extra-academic settings during adolescence may create problems to symptomatologic domains that were sufficiently compensated in childhood. However, this interpretation should be considered as purely speculative because our data is not longitudinal.

It should also be noted that additional conditions related to neurodevelopment can significantly impact on functioning. Developmental language delay is one of these conditions, and it is frequently comorbid with ADHD. However, in our sample, developmental delays (developmental language delay, developmental coordination delay) have a composite prevalence of 17.35% in subjects < 11 years and 12.26% in subjects > 11 years, and they do not significantly differ between the two groups. Indeed, this prevalence is lower than usually reported (around 30–40%) (25, 26), although some studies found a wide range (10–59%) of Developmental Language Delay prevalence in ADHD (27). Thus, it was not possible to study the potential impact of developmental delays on functioning in our ADHD sample. Longitudinal observational comparison studies between ADHD with or without comorbid developmental delays might help clarify whether these two groups share qualitatively similar developmental trajectories but different levels of impairment, or they rather display qualitatively different developmental trajectories.

The second main finding is that ADHD symptoms’ scores (CPRS-R:L, DSM-IV Inattentive and DSM IV Hyperactive/Impulsive subscales) were found to be significantly higher in the ADHD/primary school group as compared to the ADHD/secondary school group. Our results are thus consistent with the typical trajectory of ADHD-specific symptoms, usually showing a gradual decline in the total number of symptoms and/or a severity reduction for certain symptoms’ domains (2, 23, 28).

Our two main findings reported so far (i.e., less ADHD-specific symptoms and poorer functioning in older patients) could be explained in terms of a particular developmental model called “cascading anomalies.” On the base of this model early symptoms of ADHD “*per se*” may exacerbate or even create downstream neural anomalies during the developmental trajectory, so that the presence of pre-existent ADHD symptoms engenders neural dysfunction that in turn exacerbates future symptoms, including general psychopathology, and functioning problems (12). In this regard, there is substantial evidence supporting significant neurodevelopmental changes in the brain of children and adolescents with ADHD as they progress to transition into young adulthood. For example, protracted cortical thinning of the whole cingulate cortex and a progressive surface area reduction in the ventral striatum (29, 30) could represent neurobiological underpinnings of symptomatic evolution and functional worsening in terms of impaired decisional processes, emotional dysregulation and vulnerability to addiction. Conversely, a progressive improvement of ADHD symptoms is probably the result of a cortical morpho-functional restructuring in terms of a more efficient activity of the prefrontal cortex and a less pronounced interference of the default mode network on the task-dependent networks (31).

From the clinical-psychopathological point of view, emotional, social, and other internalizing aspects as somatic complaints, can be induced by other reasons not specific to ADHD, or by psychological peculiarities within the individual developmental trajectory, and may not need clinical attention “*per se*.” However, this aspects can mask underlying ADHD-specific symptoms, thus making them more difficult to detect and inducing clinicians to underestimate them (32–34). In this context, ADHD-specific symptoms during the transition to adolescence and young adulthood might be “masked” or changed but not really reduced, as confirmed by longitudinal data (23).

This leads us to the last main finding of our study, that is, the two age groups are characterized by significant differences in the emotional and behavioral profiles as measured by CBCL subscales. Specifically, the ADHD/secondary school group showed higher levels of anxious and depressive symptoms, somatic complaints, social problems, and aggression. In terms of psychopathological trajectory, we reckon that these finding parallels functional worsening, being potentially related to the adjustment process to an increase of academic and extra-academic demands. In this regard our results are also consistent with the higher risk of developing mood disorders in ADHD during adolescence (35), especially in presence of more severe externalizing symptoms and social problems in childhood (36). Consistently, CBCL-derived DP scores also differed between groups, with the ADHD/secondary school group displaying higher levels of emotional dysregulation as compared to the primary school group. DP has been correlated to poor emotional and behavioral self-regulation, it significantly predicts the development of mood disorders in adolescence, and it showed a significant association with severe psychopathology and poor adjustment (37).

Our results also confirm what Lau et al. (38) recently found on a large sample using CBCL measures. In fact, in their study, adolescents with ADHD had more internalizing and externalizing problems than children (39). However, they reported higher Attention Problems in terms of CBCL subscale scores in adolescents while we found no significant difference between our two groups on the same subscale. We interpret this inconsistency as a result of the peculiar dichotomization adopted in that study. In fact, the whole sample was split in two age groups (6–12; 13–18), and in the 13–18 group all subjects received their first ADHD diagnosis in adolescence. Thus, a longer duration of untreated ADHD symptoms in these subjects may have led to a greater persistence and severity of inattentive symptoms.

Finally, ADHD/primary school and ADHD/secondary school also differed for ongoing MPH treatment, and for first MPH prescription. In fact, it is completely reasonable to expect that the younger patients’ group displays a higher number of first MPH prescriptions and, conversely, that older patients’ group displays a higher number of ongoing pharmacological treatment. In this regard, our results are consistent with the usually reported mean age of MPH first prescription around 9 years (40). In addition, as previously stated for the diagnostic process in general, in Italy MPH treatment is still frequently delayed or under-prescribed although this has been rapidly changing over the last 10 years (41, 42).

Our study has some limitations: first, the study is cross-sectional and this limits the possibility to draw inferential conclusions with a good validity and reliability from a developmental point of view.

Second, it can be argued that more sophisticated tools can be used to assess general psychopathology as compared to the CBCL that we use here. However, it should be noted that CBCL still represents a cost-effective psychodiagnostics tool that has been shown to be valid at characterizing the types of psychopathologic conditions driving child psychiatry referrals (39).

Third, our sample was divided into two groups who were first diagnosed at different ages, and in this regards it should be considered that children needing clinical attention at earlier ages might represent more severe cases. However, it should be also considered that in Italy a first ADHD diagnosis is often made later than expected due to cultural factors like stigma (43).

Fourth, data from normal controls (i.e., age-matched typically developing subjects) are not available in our sample. For this reason, it is not possible to make reliable considerations on psychopathological evolution after age 11 as assessment after that age can be prone to confounding factors due to puberty. However, it should be also noted that clinical measures used in this study are all based on scores that are standardized on the typically developing general population.

Fifth, there is growing evidence supporting a significant association between SARS-CoV-2 related restrictions and contextual-familiar aspects impacting on ADHD symptoms’ severity, along with a significant increase in general psychopathology, especially in adolescence (44–49). Our results might have been influenced at least in part by this aspect, as part of our sample was assessed in 2020.

Finally, we acknowledge that our paper reports on a much-studied topic in ADHD; for this reason, its originality for readers may be considered limited.

Despite these limitations, we reckon that our study has the strength to provide significant and rich clinical information on a very large sample of children and adolescents with ADHD. Taken together, our results confirm a higher level of general psychopathology in adolescents with ADHD as compared to children, along with a higher severity of overall ADHD-specific symptoms in children. More importantly, older patients displayed lower levels of global functioning and poorer adaptive skills despite lower ADHD-specific symptoms’ levels. Functioning could thus remain at a low level despite less symptoms, and functional compromise could worsen due to age-related increased demands. In coping with this condition, general psychopathology phenomena might be triggered or worsened.

Indeed, our results should be interpreted cautiously as they lack longitudinal structure and data from healthy controls. Further studies with longitudinal designs and including data from age-matched typically developing subjects are needed in order to clarify whether the developmental trajectory of ADHD between childhood and adolescence is characterized by functional worsening, paralleled by an increase in general psychopathology, and a reduction of ADHD-specific symptoms.

Although these results need further confirmation, they preliminarily suggest something that should not be overlooked by clinicians in order to properly tailor multimodal treatment strategies to patients. Specifically, pharmacological treatment and/or more intensive multimodal rehabilitation treatments may be needed in older patients even in presence of a less severe ADHD-specific symptomatology, in order to tackle significant functional impairment and co-existing psychopathology.

## Data availability statement

The data presented in this study are available on request from the corresponding author. The data are not publicly available due to privacy and ethical restrictions.

## Ethics statement

The studies involving human participants were reviewed and approved by Ethics Committee of the Bambino Gesù Children’s Hospital (2541_OPBG_2021). Written informed consent to participate in this study was provided by the participants’ legal guardian/next of kin. All procedures were in accordance with the ethical standards of the institutional and/or national research committee and with the 1964 Helsinki Declaration and its later amendments or comparable ethical standards. The study was approved by the Ethics Committee of the Bambino Gesù Children’s Hospital (2541_OPBG_2021). Data were retrospectively selected and completely de-identified at the time of the study. The privacy rights of human subjects were always observed.

## Author contributions

PD, DM, and SV: conceptualization. PD, BD’A, DM, and SV: methodology. SV and DM: supervision. PD, BD’A, IP, DM, SD, and SV: writing—original draft. PD, BD’A, and DM: writing—review and editing. All authors writing—review and editing, read, and agreed to the published version of the manuscript.
